# How and why does aging occur? Updating evolutionary theory to meet a new era of data

**DOI:** 10.1093/emph/eoaf040

**Published:** 2025-12-22

**Authors:** C Jessica E Metcalf, Rozalyn M Anderson, Michael E Hochberg, Joanna Masel, Jacob Moorad, Daniel E L Promislow, Shripad Tuljapurkar, Noah Snyder-Mackler

**Affiliations:** Department of Ecology and Evolutionary Biology, Princeton University, Princeton, NJ, USA; Department of Medicine, SMPH, University of Wisconsin, and GRECC, William S. Middleton Memorial Veterans Hospital, Madison, WI, USA; ISEM, Université de Montpellier, CNRS, IRD, Montpellier, France; Santa Fe Institute, Santa Fe, NM, USA; Ecology and Evolutionary Biology, College of Science, University of Arizona, AZ, USA; Biological Sciences, University of Edinburgh, Edinburgh, Scotland, UK; Jean Mayer USDA Human Nutrition Research Center on Aging, Tufts University, Boston, MA, USA; Department of Biology, Stanford University, Stanford, California, CA, USA; School of Life Sciences, Center for Evolution and Medicine, Arizona State University, Tempe, AZ, USA

**Keywords:** aging, updating, evolutionary, theory

## Abstract

Our ability to define the causes of aging could enable targeted interventions to extend healthspan. Classical evolutionary models based on individual age have provided critical insights into empirical trajectories of aging; however, gaps remain. We argue that technological advances in data capture, resolution, and scale present a rich opportunity to shed light on heterogeneity in patterns of aging. Computational and data analysis advances have produced expanded theoretical models that explicitly address details of the underlying biology, introducing variables and dynamics that go beyond ‘age’ itself. We argue that by incorporating richer biological detail to create more integrative predictive models, we can gain insight into expected future distributions of aging within populations, and better understand the molecular and demographic context in which selection has given rise to variability in aging. We provide an overview of existing models that address heterogeneity, and outline future directions and applications that would advance this key area in aging and biomedical research.

## BACKGROUND

**Figure 1:**
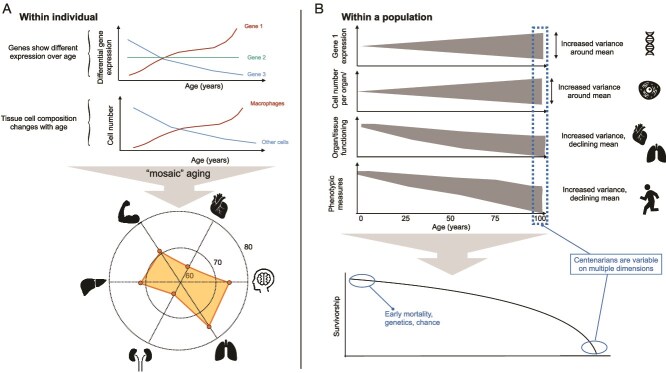
Heterogeneity in aging. (A) Aging varies at multiple levels within individuals (e.g. gene expression [1, 2]; cell functioning as single cells age [1], and epigenetic information over age [3]). These age trajectories will combine to generate ‘mosaic aging’ [4], where distinct organs show discordant biological ages (illustrated here with a radar chart for a 70-year-old). (B) Aging also varies among individuals within a population, with variance amplified by environmental exposures, gene–environment interactions, cellular processes, and, in the case of immune function, somatic generation of genetic diversity [5]. While there are some factors that appear to increase the likelihood of attaining centenarian status, as extrinsic and intrinsic factors accumulate through life, the influences on centenarian survivorship (dashed box) will thus be highly variable [6].

Evolutionary theory predicts that aging will occur, explaining why and under what circumstances; however, evolutionary theory tells us surprisingly little about how aging will actually happen. Aging is a complex biological process with remarkable variation both within and among individuals (Fig. 1). Even comparing genetically identical individuals raised in the same environments, we observe a striking degree of heterogeneity, suggesting that chance plays a significant role in this variation [7–10]. Independent evidence from the recent explosion of medical and epidemiological data points to a range of causal factors (e.g. [11]). Yet, a vast gap remains in our ability to translate this level of detail to models that can predict individual risk of age-specific morbidity or mortality. Furthermore, while there is a rich body of work on specific measures, most focus on ‘hallmarks of aging’ [12], including the molecular age-associated heterogeneity in gene expression [1], epigenetic dysregulation [3], and immune function [13] (Box 1). To date, few researchers have developed models that integrate molecular and physiological processes within a population dynamics frame to predict aging phenotypes, but see [14, 15].

Box 1. Dimensions of heterogeneity in aging: recent empirical examples.

To test theoretical models of aging that aim to meaningfully explain heterogeneity within populations, we need to quantify aging at multiple levels—among individuals, within individuals, and across time. This is a particularly challenging task because it needs to be quantified within and among organizational (e.g. molecular and cellular processes, organs, etc.) and temporal scales. The recently updated ‘Hallmarks of Aging’ [12] provides a very useful framework for understanding cellular processes that contribute to aging, and the ‘Hallmarks of Health’ [16] describes physiological decline and loss of function relevant to aging and age-related disease. Several studies have identified features that change with age and are the basis for a variety of biomarkers or so-called ‘biological clocks’ [17]. The primary application of most current molecular clocks is to predict the pace of aging and associated risk for poor health [18]. A secondary goal is to elucidate the causal mechanisms behind these clocks, though this will likely require different tools and different conceptual frameworks. Others have begun to titrate the roles of dynamic processes. For instance, selection on the mitochondrial genome counteracts the age-related accumulation of damaged molecules that precipitate damaging intracellular signals [19].

Importantly, recent technological advances and longitudinal datasets now provide both the data and resolution needed to benchmark and test these theoretical models. Single-cell and spatial transcriptomics enable quantification of how cell states and tissue microenvironments differ over the life course–providing cellular and sub-cellular resolution of within- and between-individual heterogeneity. Longitudinal multiomic datasets—including DNA methylation, proteomics, and metabolomics—are becoming more abundant [20], which allows us to identify the molecular pathways underlying differential aging trajectories. High-throughput imaging and wearable sensors capture functional decline at the tissue and organismal level in real time, linking molecular changes to physiology. Population-scale biobanks and large-scale comparative studies (e.g. the UK Biobank, Dog Aging Project, and All of Us) now provide the sample sizes and diversity needed to model how genetics, environment, and chance jointly shape health and aging.

Identification of factors that causally contribute to heterogeneity in how aging manifests among individuals necessarily requires a more expanded and integrated approach. In general, it is reasonable to assume that not all variables are sensitive to age, nor would changes induced by age be equivalently observed among variables. Some age-related changes may be adaptive rather than detrimental, or even vary in their effect on fitness across the lifespan (e.g. antagonistic pleiotropy), and thus be unsuitable as targets to delay aging. Further complicating the matter, heterogeneity in health and longevity may not be equivalent across phases of the lifespan. Essentially, the variables that afford us individuality also pattern our heterogeneity in health and in aging; it seems ‘one size’ will not fit all. On the positive side, identifying the right size for each of us should not only be within our grasp but should have us all looking good.

**Figure 2:**
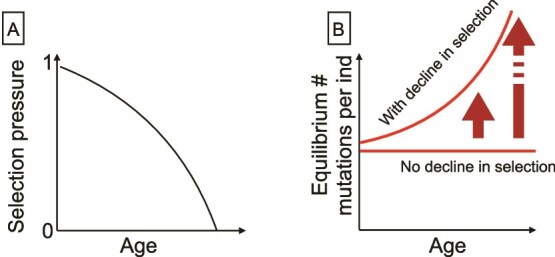
Basic evolutionary expectations under a very simple model. (A) Following standard results from evolutionary theory [24], selection pressure (y axis) declines with age (x axis), and phenotypes expressed at late ages fall within the ‘shadow of selection’. (B) As a result, assuming that there are germ-line mutations with strictly age-specific effects, we expect the accumulation of late-acting mutations, ultimately leading to mortality cliffs at the point where selection pressure is zero. Modifications of the underlying framework, such as altering the mapping from mutations to mortality, can alter this outcome (see text).

Despite considerable variation among individuals within species, we observe strikingly general properties across species. In many species and in many settings, all-cause and disease-specific mortality rates increase exponentially with age in a consistent fashion [21]. The broad distribution of these patterns is not explained by current theory from evolutionary biology. While such mortality dynamics emerge from some evolutionary models [22], it is also relatively straightforward to develop models where mortality rates do not increase exponentially with age [23]. Indeed, the simplest evolutionary theory predicts that mortality risk should rapidly transition to near infinite after reproduction ceases [24, 25], translating into negligible heterogeneity in the timing of death [26]. However, slight modifications to the underlying framework, e.g. adding complexities such as variance in the age-specific effect of mutations [27] and nonlinearity in the effects of mutational accumulation [25], can replace this ‘wall of death’ (Fig. 2) with a mortality plateau ([24, 25]).

These examples illustrate the limits of minimalist models in terms of the underlying biology. Early evolutionary genetic models of aging simply assumed that germline mutations translate directly to increased (or reduced) mortality risk. While such models still yield important insights (e.g. only recently has theory shown why we might expect different traits—and also in principle, different tissues or organs—to age at different rates [28]), there are inevitable limitations to what simple models can deliver. A more comprehensive approach will be necessary to illuminate how aging will occur and may allow for the generation of valuable predictive models. Here, we suggest that a focused effort to reconcile the vast heterogeneity in aging with evolutionary theory has the potential to lay the foundation for theoretical and empirical research and yield new insights into both the ‘how’ and ‘why’ of aging.

Researchers have developed a range of conceptual and theoretical descriptions to reflect the underlying mechanisms of aging, including: (i) a fundamental role for resource allocation among processes contributing to maintenance, growth, and reproduction [29] (albeit often extremely hard to measure); and (ii) that aging might emerge as an inevitable side-effect from the challenges of restructuring complex system processes during the transition from the developmental to adult phase [30]. Many of these models remain largely data-free, yet considerable applicable data exist (Fig. 1, Box 2). Furthermore, models rarely address how individual age trajectories are shaped by a range of potentially important ecological factors, such as resource availability (nutrition [31]), pests, or pathogens (immunity; e.g. [32]), the social context [33], climatic stressors (e.g. shifting temperature, natural disasters; [34]), and so forth. More nuanced

Box 2. Modeling to meet heterogeneity in aging.

Model construction is usefully guided by the old adage ‘as simple as possible but no simpler’. The scale of simplicity that is appropriate is shaped by the focal question. In the field of aging, we can broadly distinguish between three distinct ways in which models are used:

1. Models that predict aging phenotypes using expectations from life history theory to frame hypotheses around physiological processes and tradeoffs; diverse biological measures are then integrated within a dynamic frame to predict patterns of aging [14, 15]. The domain of application of such models remains an open question—e.g. would models developed for worms [35] apply to mice? Comparative frames leveraging cross-species or between-sex genetic underpinnings of longevity [36] or cross-species patterns of rates of aging [37] provide insights that further steer the development of such models.
2. Models that produce empirically testable predictions capable of distinguishing among previously verbal hypotheses, e.g. regarding which specific traits exhibit trade-offs that shape evolutionary optimization [38, 39].
3. Models that provide proof (or disproof) of principles regarding how aging phenotypes *might* emerge [40], expanding on a scaffolding of hypothesized tradeoffs to ask how evolution is expected to proceed [28]. For example, this type of analysis has been developed to reveal how ‘calorie restriction’ could emerge from fitness maximization if resource availability is variable and there are resource thresholds for successful reproduction in mice [41].

How these models are constructed will be contingent on the blend of known mechanisms and data available. For example, for the third type of model, phenomenological models constructed around features like size may suffice. While some model assumptions are ‘critical’ to the principle being proved or disproved, other assumptions are merely ‘exploratory’ or ‘logistical’ [40], and can thus remain coarsely characterized. Semi-mechanistic models may prove powerful in this instance—some elements of the model will be explicit, and others reflect more of a ‘black box,’ which could be captured reflecting phenomenological processes of varying complexity, from simple descriptions to complex multivariate machine learning inputs, depending on the scale of available data.

models of the underlying biology have the potential to shed light on the striking plasticity in healthspan and lifespan, as exemplified by almost two centuries of steady increase in life expectancy in some human populations [21].

In this perspective, we consider opportunities for advancement in aging theory, challenges in integrating detailed data on heterogeneity of aging within an eco-evolutionary framework, and ways in which an evolutionary perspective could predict the emergence of heterogeneity in aging. We anticipate that this will be of interest to: (i) population biologists and geroscientists by providing a framework that unifies the comparatively simple models of aging with the rich and growing collection of biological data; (ii) experimental biologists by providing ways to identify model systems where specific aspects of aging heterogeneity should (or should not) be modeled; and (iii) gerontologists and geriatricians by identifying and quantifying the relative importance of intrinsic and extrinsic factors on aging trajectories to inform both precision prognosis and intervention.

## INTEGRATIVE MODELING FOR THE BIOLOGY OF AGING

### Models that aren’t all about age-specific mutations

As noted above (Fig. 2), the foundational evolutionary models of senescence argue that aging evolves due to an age-related decline in the force of selection, which in turn leads to the accumulation of late-acting deleterious mutations [24, 42]. The age-specificity of mutations that increase mortality risk at later ages can now be directly measured empirically [43]. This exercise reveals considerable variance in age of onset of mortality-associated mutations [27], and framing these empirical patterns of mortality associated with mutations within demographic models reflecting human mortality and fertility trajectories indicates that many known mutations associated with late-onset genetic diseases are under purifying selection. This signature of selection could be due to factors that extend the force of selection into ages beyond what is expected by standard models, such as with grandparent effects [44], due to different sex-specific mating patterns across ages [45], or due to the case where effects of mutations are positively correlated across ages [27, 46], i.e. indirect selection favoring post-reproductive life-span emerges from early/late pleiotropy [44]. While biological features occurring between mutation and phenotype, such as processes of growth or the development of the immune system, are not explicitly considered, by including co-variance of mutational effects across age in mapping from mutation to mortality, such models start to reflect some of the complex processes by which mutations lead to the emergence of specific mortality patterns [47].

### Models that address within-individual dynamics

Within any multicellular organism, cellular dynamics will vary within and across individuals. Damage in a single cell can be passed along to daughter cells, leading to clones with reduced fitness [48]. Natural selection can select for mechanisms that slow the rate of cellular degradation, but degradation cannot be completely arrested. Within-individual competition might eliminate these malfunctioning cells to the benefit of the individual organism [49]. Conversely, competition might favor ‘cheating’ cells that preferentially survive and replicate, resulting in clonal expansions [50] (or, in the worst case, cancer [51]), to the detriment of the organism. This presents two distinct trajectories of aging, one in which cells become less vigorous, another in which they retain cellular vigor but contribute less to the healthy functioning of the organism [49].

‘Cellular senescence’ is one way in which cells might become pathogenic: this response to stress or damage entails cells entering cell cycle arrest that is concomitant with the production of pro-inflammatory secreted factors. Pathology arises when these stalled cells are not cleared but persist within the organism. Continued presence of senescent cells markedly reduces organ function, but it also potentially reduces the number of cell divisions occurring in the organ as a result of homeostatic control of organ size [52]. A reduced number of cell divisions reduces both the occurrence of and selection on somatic mutations (and epimutations), mitigating the risks of cell death and cancer, respectively. The resulting tradeoff between negative outcomes from somatic mutation and negative outcomes from reduced organ function could also shape the evolution of cell senescence [53, 54].

Just as cell–cell interactions within an individual can influence the aging trajectory, so too could interactions among microbes within an individual. That is, within-host competition could play out at the scale of the microbiome, the microbial organisms that live in and on us. The microbiome is increasingly recognized to shape our health, and among individuals? becomes more heterogeneous with age [55], making it an attractive subject for further modeling. These various cellular processes and their heterogeneity will also translate into heterogeneity in phenotypes (size, etc.). Below, we introduce phenomenological models of such phenotypes, but note that there is considerable potential in developing empirically derived models that link the two.

### Models that include heterogeneity in phenotypic trajectories

**Figure 3:**
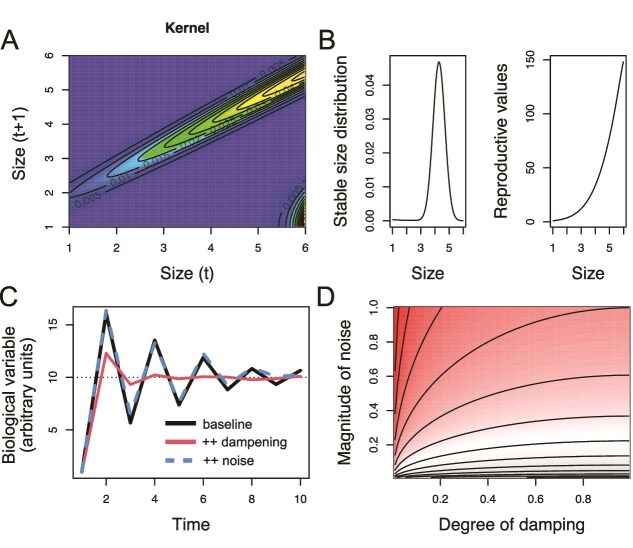
Examples of models that introduce forms of heterogeneity, illustrating both an existing technique focusing on phenotypic heterogeneity in size (top row) and an area under development, i.e. probing the implications of homeostatic systems for aging (bottom row): (A) *an integral projection model, or demographic model capable of including individual variance into individual trajectories.* In this example, size at time *t* (x axis) maps to size at *t + 1* (y axis) along the main diagonal as a result of growth; the peak in the lower right corner reflects reproduction: Individuals at large sizes at time *t* (x axis) produce small individuals at *t + 1* (y axis). The spread around the diagonal captures individual variance in growth, which means that individuals of the same age may be very different sizes. Colors indicate transition, with shading toward yellow indicating higher transition probabilities*.* (B) from this demographic frame, we can predict the stable size distribution and reproductive value, i.e. both ecological and evolutionary outcomes; (C) *a model reflecting a homeostatic system.* A biological variable *G* starts far from its equilibrium ${B}_{eq}$ and then changes following damped oscillations according to ${G}_{t+1}={G}_{eq}-\beta \left({G}_t-{G}_{eq}\right)+\sigma{\epsilon}_t$ where $\beta$ reflects a damping ratio (lower $\beta$ translates to greater damping), and $\sigma$ represents the noise effect. Three trajectories are illustrated: A baseline (black line), the same with an increase in damping i.e. reduction in $\beta$, resulting in a faster return to equilibrium (red line), and as baseline but with an increase in the magnitude of the noise effects, resulting in large, erratic perturbations (blue line). (D) if we assume that mortality scales with distance from equilibrium in a homeostatic system (here set to ${G}_{eq}=10$), then mortality is inversely related to damping ($1-\beta$, x axis) or $\sigma$ (y axis) on the surface plot (risk of mortality here indicated by darker shades of red), as the variance in ${G}_t$ is defined as ${\sigma}^2/\left(1-{\beta}^2\right)$.

Rather than focus on genetics and the mutations underlying aging, an alternative possibility is to focus on phenotypes that influence the fundamental components of fitness–age-specific fertility and mortality—(e.g. physiological maintenance, reproductive effort, resource allocation, regulatory phenotypes). One example of this comes from integral projection models [56] that include combinations of statistical models of growth, survival. and reproduction in structured demographic models. This framework allows stable population structures, fitness, reproductive values, etc. to be extracted. Critically, these models can include the ubiquitous observation of variance in growth (Fig. 3A), alongside structuring by myriad biological features (age, size, quality, immune status, etc.), providing a much more nuanced prediction of selection landscapes [57]. Phenotype-scale phenomenological modeling of processes such as recovery from infection has the potential to yield new insight into individual trajectories and patterns of selection [58]. However, while these types of approaches would afford an important advance on models of selection structured solely around age, characterization remains at the scale of the phenotype, and ignores details of the underlying physiology and/or genetic architecture and environmental dependencies that can importantly shape lifespan [59]. Additionally, such approaches often leave out underlying feedback that could substantially alter host aging and health outcomes in subsequent years. For example, inflammation driven by immune responses to infection is often swiftly down-regulated, but the specifics of how this happens are sensitive to initial conditions or context and can substantially influence outcomes [60]. As a result, near-identical individuals can have strikingly different health outcomes (i.e. the dynamical regime is characterized by alternative stable states). Purely phenomenological models will not capture this possibility, making similar predictions for all individuals.

### Models that include within-organism feedback mechanisms

A growing body of research has led to an increased understanding of the complex role of feedback in biological systems. These feedback systems are central to the maintenance of homeostasis, as is simply and powerfully illustrated by the role of insulin in glucose homeostasis. Insulin is secreted in response to glucose elevation and impacts glucose uptake, energy storage, and anabolic pathways in target tissues. Once glucose has been cleared, insulin recedes from circulation, and those processes are halted. The importance of this pulsatility is evident in its failure, leading to the onset of insulin resistance, impaired fasting glucose, and the eventual transition to diabetes. Despite the key role that homeostatic systems play in health and mortality risk [61] (Fig. 3B), they are rarely considered in evolutionary models of aging (barring some notable exceptions [62]). Particularly striking results can be obtained in models of immune system functioning where feedback is very strong, and can lead to the emergence of alternative stable states [63]. The next step in modeling will be to link established feedback mechanisms (e.g. those with associated insulin function, patterns of immune reactivity, etc.) to realistic environmental perturbations and their interactions with other parts of the system. Such an integrative approach would reveal new perspectives on the ‘shadow of selection’ and how it influences underlying mechanistic drivers of homeostasis and should yield important insights into the heterogeneity of aging as an outcome.

### Models that include explicit responses to the environment

How individuals respond to their environment has strong implications for health, aging, and mortality. In general, models do not often formally include such responses to environmental variables but focus instead on other well-described phenomena ranging from bet-hedging (or ‘intra-genotypic variance’) to phenotypic plasticity or aspects of biological systems that reflect ‘long-term programming’ [64]. Taking the examples of cognitive systems or immunity, it is well established that there is an early life window or ‘critical period’ associated with ‘learning’ during which later life patterns are established. Environmental predictability is a critical aspect of the degree to which such patterns might evolve [65]; more predictable environments across longer generation times will lead to fixed strategies with less flexibility among individuals, while less predictable environments that change over shorter timescales will likely lead to more variable phenotypes [65]. In some instances, the fitness advantages of particular aspects of heterogeneity are well described, including diversified bet-hedging via variable timing of seed germination or flowering [66], but this remains relatively rare. Although this framework can provide valuable insights into how individuals respond to environmental variables, it provides limited insight on connections to broader processes of aging, such as aging mechanisms, intricacies of human systems, such as sociocultural factors, and within-lifespan variation in environmental responsivity and resilience [67]. Extending the analytical framework to environmental responses to the biological, social, and cultural dimensions of aging has the power to enrich our understanding of how adaptive strategies shape aging dynamics and influence outcomes in diverse environments.

## AN INTEGRATED PERSPECTIVE

The time is ripe to move from considering a single dimension (e.g. chronological age) in modeling aging and its evolution to models that incorporate the molecular and pathophysiological features for which we now have empirical measures (Box 1). Major challenges still lie ahead. Despite decades of advances, we have a relatively limited understanding of the relevant biology underlying heterogeneity in aging and how dynamics and feedback loops contribute to these complex outcomes. A variety of first steps have been taken, as detailed above. Continued progress, building on these first steps, set the stage for full integration of complex biological models within a larger evolutionary framework. A key feature of these new developments is the emphasis on biology, but not simply via inclusion of a larger number of variables. Rather, the goal should be on the identification and incorporation of elements that address clearly articulated hypotheses. In this way, one idea at a time, it should be possible to connect aging, morbidity, and mortality. The culmination of these efforts should illuminate how those core elements change, why they change, and under what circumstances.

**Figure 4:**
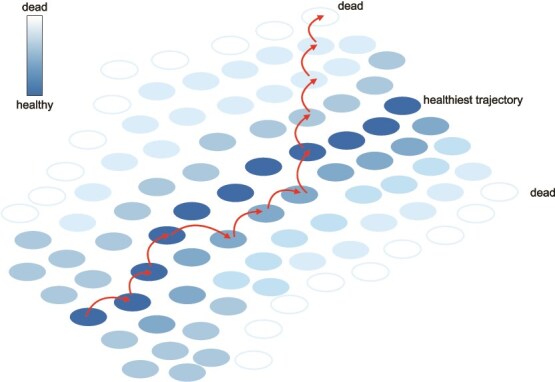
A conceptual diagram reflecting heterogeneous trajectories of aging. Each ‘lily pad’ reflects a different relative state of health (defined by a wide array of different variables, size, social context, biomarkers, etc.), with darker pads indicating a specific set of healthy trajectories. In every time step, individuals shift to the next lily pad, with perturbations potentially pushing them off the healthy trajectory (fourth jump), or engaging homeostatic and restorative mechanisms that allow them to return to good health following a setback (7th jump). The ability to return to health is expected to decline at late ages, influencing shift direction probability, and may reach a point of no return that leads to the eventual demise of the individual. For any trajectory, lily-pads eventually fade to white in a context of senescence. Note also that variation among trajectories is distinct from variation in timescale–the latter can involve exactly the same series of jumps occurring at different speeds.

The models we introduce here grapple with different processes, from temporal trajectories to feedback mechanisms to responses to the environment, all of which happen in all individuals. Organizing our thinking around these models points to the need for a larger conceptual model of how we define and consider aging. Individuals move through trajectories across multiple dimensions, including time/age, size, behavior, exposures, injury, and disease incidence. Although we would note that there are probably many ways to be ‘healthy,’ a useful analogy is a frog jumping across lily pads in a bounded space reflecting aspects of health (Fig. 4). Movement is shaped by current and past states, successive leaps are governed by chance, environmental exposures and individual responses to them, as well as by underlying processes such as feedbacks associated with homeostasis. A range of approaches is possible to construct a model that reasonably reflects this ‘path across the lily pond’. Rather than reducing dimensionality of the system (e.g. [14]), by opening the door to efficient calculation of the distribution of possible fates across multiple measures, methods such as adaptive integration [68] enable researchers to accommodate the full set of variables (size, age, biomarker density, etc). Insistence on maintaining age within the model retains short-term path dependency with adjustments made as necessary for differences in time between transitions and the directionality (toward healthier or less healthy) associated with each leap. Complexities within these frames associated with biological processes, such as underlying feedback, may also be considered, allowing a dynamic, adaptive, and fully integrated set of permutations (alternative paths and investments) to be evaluated. One interesting concept that emerges from this framing is that the variety of stressors that individuals inevitably experience over their life course will result not only in heterogeneity in observed trajectories of aging, but also in selection for different, possibly variable responses, from plasticity to bet-hedging that must be considered alongside the footprint of ‘priority effects’—or long-term impacts of early life stressors. We believe this feature is general across biological systems and merits concerted attention.

Progress toward an integrative framework will depend on connecting theoretical abstraction with empirical data across organizational scales. Advances are likely to come from models that can be parameterized and experimentally validated in tractable systems such as *Caenorhabditis elegans*, *Drosophila*, and *Saccharomyces cerevisiae*, where molecular, phenotypic, and population data provide the necessary elements to link mechanism to observation [69]. Although highly challenging, insights from these and other systems can then be scaled up and cross-validated using long-term ecological and demographic datasets from natural populations, as well as clinical and biobank data that capture human heterogeneity in genetics, environment, and lifestyle [15]. The interplay between data-driven and theory-driven approaches will be key—theoretical models can guide exploration of parameter space and assess relative influence, while empirical datasets would provide bases for parameter estimation and simulating heterogeneity in aging. Such an approach offers a realistic route toward predictive and testable models that capture the diversity of aging trajectories and tests of evolutionary theory.

## CONCLUSIONS

Theories of aging have been limited in their ability to explain variation in individual aging trajectories. Integrating biological heterogeneity into evolutionary theory gives us the power to address the complexity of aging both within and among individuals, which will substantially improve our understanding of aging and its health correlates. Doing so requires frameworks that extend beyond relatively simple models of age-specific mutations to include dynamic feedback, phenotypic plasticity, and environmental responsiveness. Moreover, these models have the power to uncover the mechanisms underlying variability in aging trajectories and to develop more precise predictions of age-associated outcomes. We believe these models hold immense promise for bridging gaps between experimental biology, biodemography, and biogerontology, paving the way for transformative insights into the mechanisms, causes, and consequences of aging. The time is ripe for us to refine theoretical frameworks that will guide empirical research and contribute to innovations that address fundamental and applied challenges in geroscience and aging biology. The recent and rapid spread of GLP-1 agonists, which not only facilitate weight loss but also appear to ameliorate the risk of multiple age-related diseases [70], while also having some negative effects (e.g. muscle loss [71]), gives us a glimpse into what the future might look like. Considerable uncertainty about long-term outcomes of these types of interventions [72] amplifies the societal relevance and urgent need to address these questions.
